# An unusual case of left venous renal entrapment syndrome: a new type of nutcracker phenomenon?

**DOI:** 10.1007/s00276-012-1027-7

**Published:** 2012-10-02

**Authors:** Michał Polguj, Mirosław Topol, Agata Majos

**Affiliations:** 1 Department of Angiology, Medical University of Łódź, Narutowicza 60, 90-136 Łódź, Poland; 2 Department of Normal and Clinical Anatomy, Medical University of Łódź, Narutowicza 60, 90-136 Łódź, Poland; 3 Radiology Department, Medical University of Łódź, Kopcińskiego 22, 90-153 Łódź, Poland

**Keywords:** Nutcracker phenomenon, Computer tomography angiography, Venous renal entrapment syndrome

## Abstract

Left venous renal entrapment syndrome was observed during multidetector 64-row computer tomography and color Doppler ultrasonography in a 58-year-old Caucasian female hospitalized due to choledocholithiasis. The patient demonstrated no typical symptoms of nutcracker syndrome. The left renal vein (LRV) was compressed as it passed between the superior mesenteric artery and the right renal artery. The LRV lumen measured 1.7 × 7.8 mm (width × height) at the point of narrowing and 7.5 × 17 mm before this. Secondary to the nutcracker phenomenon, the course of left ovarian vein was winding and was significantly wider than the contralateral vessel.

## Introduction

Left venous renal entrapment syndrome, commonly called the nutcracker phenomenon (NCP), is characterized by impeded outflow from the left renal vein (LRV) into the inferior vena cava (IVC) due to extrinsic LRV compression when passing through the angle between the abdominal aorta and the superior mesenteric artery (SMA). This pathology is accompanied by demonstrable lateral (hilar) dilatation and medial (mesoaortic) narrowing. The nutcracker syndrome (NCS) is the clinical manifestation caused by the situation that the LRV suffers from pressure when passing through the narrowing formed by the abdominal aorta and the SMA [14, 17].

The increased venous pressure within the renal circulation promotes the development collaterals of the renal pelvis, and this plexus of abnormal hypertensive veins causes micro- or gross-hematuria. Other possible symptoms include left flank pain, left-sided varicocele congestion, chronic pediatric fatigue syndrome, orthostatic proteinuria, and gastrointestinal symptoms. The most common syndromes were: hematuria or albuminuria, left flank pain, lumbar pain, and varicocele [13].

The first clinical report of this phenomenon was by El-Sadr and Mina [4] in 1950. The term “nutcracker syndrome” is usually credited to Belgian physician De Schepper [3], although it was first used by Chait et al. [2] in 1971. However, the earliest pathologic description belongs to the anatomist Grant (1937) [5].

To best of our knowledge, it is the first report of the left venous renal entrapment caused by compression created by the SMA to the right renal artery. An accurate depiction can be also valuable in the preoperative evaluation of patients undergoing donor nephrectomy, especially since newer laparoscopic techniques are now routinely employed for this type of surgery.

## Case report

A 58-year-old Caucasian female was admitted to the surgical department of our hospital for acute abdominal pain associated with vomiting. She had suffered cholecystectomy 12 years ago. A physical examination revealed palpation pain in the right epigastrium. Choledocholithiasis was diagnosed by ultrasonography of the abdomen. An endoscopic retrograde cholangiopancreatography was used to remove three gallstones.

**Fig. 1:**
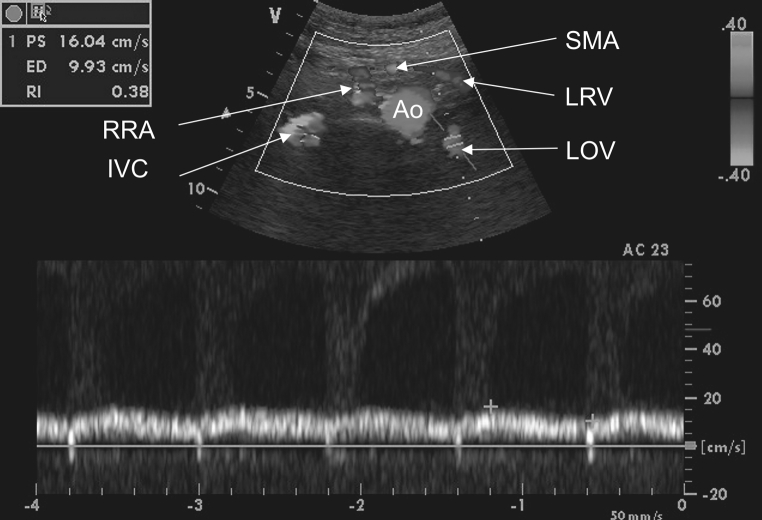
Color Doppler ultrasonography abdominal vessels. *Ao* abdominal aorta, *IVC* inferior vena cava, *LOV* left ovarian vein, *LRV* left renal vein, *SMA* superior mesenteric artery, *RRA* right renal artery

**Fig. 2:**
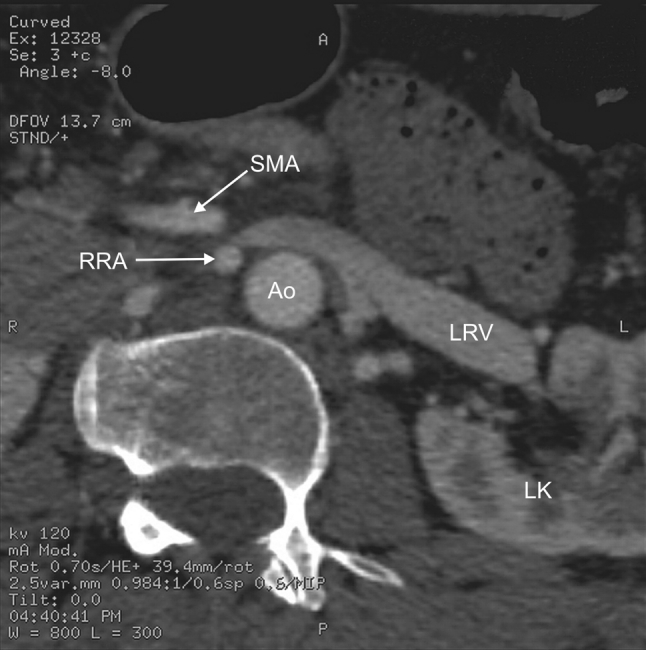
Helical CT angiography, transverse scan at the L2 level. *Ao* abdominal aorta, *LRV* left renal vein, *LK* left kidney, *SMA* superior mesenteric artery, *RRA* right renal artery

**Fig. 3:**
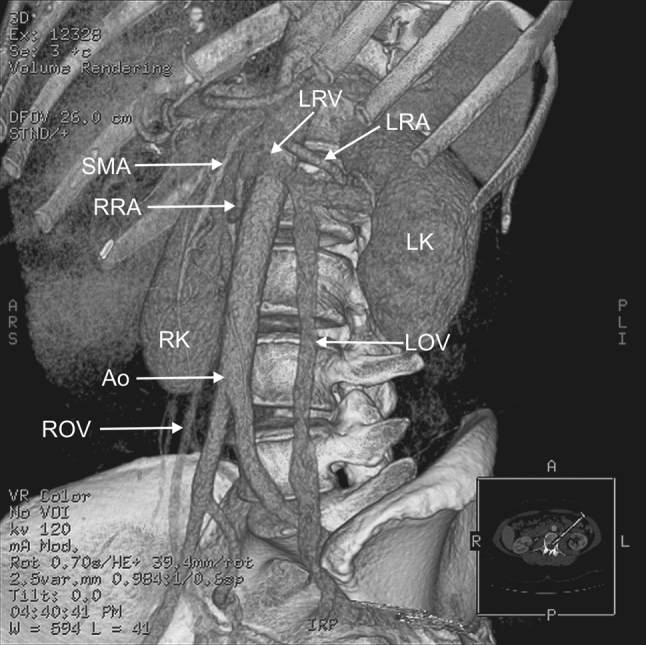
Three-dimensional CT reconstruction of the structures of the abdomen. *Ao* abdominal aorta, *LK* left kidney, *LOV* left ovarian vein, *LRA* left renal artery, *LRV* left renal vein, *SMA* superior mesenteric artery, *RK* right kidney, *RRA* right renal artery, *ROV* right ovarian vein

In addition, color Doppler ultrasonography (Vivid 7 Pro, GE) revealed that the diameter of the LRV had expanded to 9 mm (Fig. 1). Computer tomography angiography (TK-64-row MDCT scanner, LightSpeed VCT, GE, Waukesha, Wisconsin, USA) also revealed that the LRV was compressed when passing between the SMA and the right renal artery (RRA) (Figs. 2, 3).

**Fig. 4:**
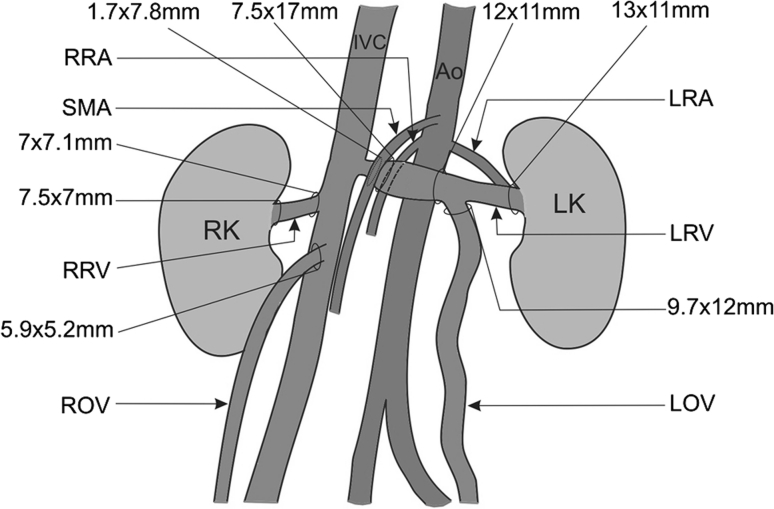
Schematic arrangements of the abdominal vessels. *Ao* abdominal aorta, *IVC* inferior vena cava, *LK* left kidney, *LOV* left ovarian vein, *LRA* left renal artery, *LRV* left renal vein, *SMA* superior mesenteric artery, *RK* right kidney, *RRA* right renal artery, *ROV* right ovarian vein

The LRV lumen decreased to 1.7 × 7.8 mm at the narrowings (always, respectively width × height) but measured 7.5 × 17 mm directly before. The shape of the LRV at the levels of the left ovarian vein (LOV) outlet and renal hilum was almost cylindrical, with average measurements of 12 × 11 mm and 13 × 11 mm, respectively. Comparatively, the lumen of the contralateral right renal vein (RRV) at the level of the renal hilum and at its opening to the IVC, measured 7.5 × 7 and 7 × 7.1 mm, respectively. The course of the LOV was winding. Its lumen before opening to the LRV was 9.7 × 12 mm. Comparatively, the course of the contralateral right ovarian vein (ROV) was almost straight. Its lumen before opening to RRV was 5.9 × 5.2 mm (Fig. 4).

**Fig. 5:**
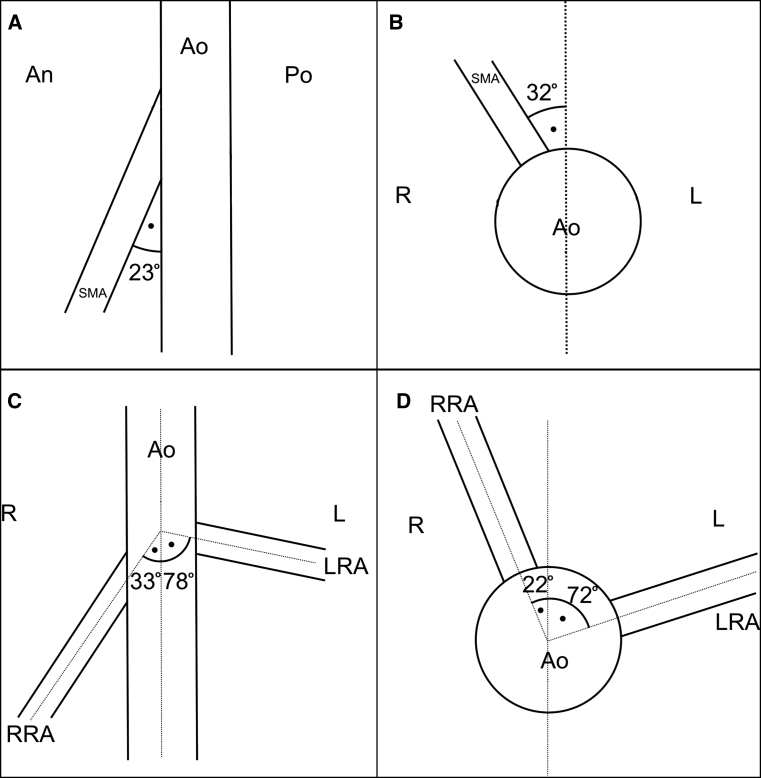
Schematic arrangements of the angles between abdominal arteries. **a** Angle between the abdominal aorta and first part of the superior mesenteric artery measured in the sagittal plane, **b** angle between longitudinal axes of abdominal aorta and first part of the superior mesenteric artery measured in the transverse plane, **c** angle between the longitudinal axes of abdominal aorta and renal arteries measured in the frontal plane, **d** angle between the longitudinal axes of abdominal aorta and renal arteries measured in the transverse plane. *Ao* abdominal aorta, *SMA* superior mesenteric artery, *RRA* right renal artery, *LRA* left renal artery, *R* right, *L* left, *An* anterior, *Po* posterior

In addition, the angles at which the SMA arose were measured. The first averaged 23° between the abdominal aorta and the first part of the SMA, measured in the sagittal plane (Fig. 5a). The second was found to be 32° between the longitudinal axis of the abdominal aorta and the SMA, in the transverse plane (Fig. 5b).

Both renal arteries arose from the abdominal aorta at the level of L2, below the origin of the SMA, with the renal vein being anterior to the renal artery. However, the arising of the RRA was atypical and demonstrated a precipitous downward course to the right kidney. Therefore, it was decided to collect angles in two planes between renal arteries and abdominal aorta to better describe this phenomenon. First was the angle measured in the frontal plane between the longitudinal axes of the abdominal aorta and the left and right renal arteries, which averaged 33° and 78°, respectively (Fig. 5c). The second was the angle measured in the transverse plane between the longitudinal axes of the abdominal aorta and the left and right renal arteries, which were found to be 72° and 22°, respectively (Fig. 5d).

## Discussion

Kurklinsky and Rooke [8] emphasize that the terms “nutcracker syndrome” (NCS) and “nutcracker phenomenon” (NCP) are sometimes used interchangeably in the literature. The NCS is the clinical equivalent of NCP characterized by a complex of symptoms with substantial variations. Also Shin and Lee [14] state that the “nutcracker anatomy” is not always associated with clinical symptoms and that some of the anatomical findings suggestive of nutcracker may represent a normal variant or be accounted for by other conditions. Therefore, the term “nutcracker syndrome” should be reserved for patients with characteristic clinical symptoms associated with demonstrable nutcracker morphologic features [13, 14].

In the recent literature, the nutcracker phenomenon (syndrome) is divided into two types: anterior and posterior nutcracker phenomenon (syndrome). “Anterior nutcracker phenomenon (syndrome)” was described as a result from compression of the LRV between the SMA and the abdominal aorta [1, 15]. In a similar fashion, the term “posterior nutcracker phenomenon (syndrome)” refers to left renal venous hypertension secondary to compression of the retroaortic LRV between the abdominal aorta and the vertebral column [1, 9].

Both types of this pathology result from subsequent development of venous varicosities of the renal pelvis, ureter, and the gonadal vein [1, 15]. The syndrome is manifested by left flank and abdominal pain, unilateral hematuria, and occasionally a varicocele in the male or abnormal menstruation in the female [10].

In our case, the patient demonstrated no typical symptoms of NCS, hence this pathology was classified as NCP. According to the localization of narrowings (antero-lateral to the aorta), it might be classified as “anterior type.” However, in our opinion, a better description would be “antero-lateral” or “lateral type nutcracker phenomenon.” This would enable the “classical anterior type,” where the LRV is compressed in the angle between the aorta and superior mesenteric artery, to be distinguished from the phenomenon reported in this study.

Physiologically, in normal condition, the pressure difference between the LRV and the IVC is less than 1 mm Hg. In NCS, it is more than 3 mm Hg, therefore, the abnormal communicating branches appear between the sinus and the renal calyces because of the venous congestion and induce hemorrhage [12].

Lopatkin et al. [10] postulate that increased pressure in the left renal and gonadal veins resulted in rupture of the thin-walled septum between the small veins and collecting system in the renal fornix. However, the pathophysiology of abnormally high pressure in these veins are not yet understood. Wendel et al. [16] hypothesize that this phenomenon may result from abnormal posterior renal ptosis with subsequent stretching of the LRV over the aorta. They speculate that stretching of the LRV over the aorta and dorsal ptosis of the left kidney in the supine position, due to lack of retroperitoneal fat, contribute to LRV compression. Other investigators have proposed that the syndrome may be due to abnormal branching of the SMA from the aorta [15].

Posterior NCP is manifested as a left venous anomaly. Here, the single renal vein follows a posterior course to the aorta and drains into the IVC. The completely retroaortic renal vein is seen in 3 % of patients [7]. However, this topography of the LRV is not always associated with clinical symptoms, since the retroaortic renal vein can also drain into the iliac vein [7].

The left renal anatomy is especially crucial because it is the preferred side for resecting the donor kidney. Tributaries of the LRV are prominently displayed and are of potential surgical importance if noted as enlarged, particularly in the case of family donors. It is not known whether genetics have an influence on the predisposition of left renal entrapment syndrome. In the recent literature, there was one description of NCP in two siblings of a Japanese family. These patients were a 3-year-old brother and a 5-year-old sister born to healthy non-consanguineous parents. Their family history manifested no renal diseases [11]. However, every description of vascular variations in the renal region is important as they contribute toward a future meta-analysis.

According to Zhang et al. [17], the angle described in our case between the abdominal aorta and SMA measured in the sagittal plane (23°) is abnormal: the normal angle is greater than 45°. When the angle is less than 35°, it is significant for the diagnosis of NCP. Shokeir et al. [15] confirmed these findings in three patients with angles between the aorta and the SMA of 42°, 47°, and 51°, compared to the usual rectangular branching seen in 12 healthy donors. However, Ali-El-Dein et al. [1] describe the mean angle between the aorta and SMA in the sagittal CT angiography in NCP as 54°, compared to 91° in healthy controls.

Scholbach [13] recommended a diagnosis of NCP when the caliber of the LRV reduced by more than 50 % while crossing the abdominal aorta. For measurement, a longitudinal section of the vein, as well as its maximal and minimal diameter was recorded. The average normal LRV diameter is 4–5 mm [6]. In our study, as the lumen of the LRV amounted to 1.7 × 7.8 mm (width × height) on the level of compression, it complied with conditions of left vein entrapment phenomenon.

The treatment of NCS depends on age and clinical symptoms. For patients younger than 18 years, the best option is a conservative approach with observation for at least 2 years because as many as 75 % of patients will have complete resolution of hematuria. A variety of surgical approaches have been used, including medial nephropexy with excision of renal varicosities, LRV transposition with or without Dacron wedge insertion between SMA and aorta, LRV bypass, renal-to-IVC shunt, renal autotransplant, SMA transposition, gonadocaval bypass, and even nephrectomy for persistent hematuria [8, 13].

Understanding the topography and variations of the abdominal vessels is important for diagnosis, endovascular intervention, and surgery. To the best of our knowledge, the left venous renal entrapment caused by compression created by the SMA and the RRA has not been previously reported, making this case is particularly important in surgery of the renal region.
